# Fetal endothelial colony-forming cell impairment after maternal kidney transplantation

**DOI:** 10.1038/s41390-022-02165-x

**Published:** 2022-06-22

**Authors:** Nadia Meyer, Thu Huong Vu, Lars Brodowski, Bianca Schröder-Heurich, Constantin von Kaisenberg, Frauke von Versen-Höynck

**Affiliations:** 1 Gynecology Research Unit, Hannover Medical School, Carl-Neuberg-Strasse 1, D-30625 Hannover, Germany; 2 Department of Obstetrics and Gynecology, Hannover Medical School, Carl-Neuberg-Strasse 1, D-30625 Hannover, Germany

## Abstract

**Background:**

Successful pregnancies are nowadays possible after kidney transplantation but are associated with a higher incidence of maternal and fetal complications. Immunosuppressive therapy causes cardiovascular side effects but must be maintained during pregnancy. Little is known about the consequences of maternal kidney transplantation on offspring’s endothelial health. Endothelial colony forming cells (ECFCs) represent a highly proliferative subtype of endothelial progenitor cells and are crucial for vascular homeostasis, repair and neovascularization. Therefore, we investigated whether maternal kidney transplantation affects fetal ECFCs’ characteristics.

**Methods:**

ECFCs were isolated from umbilical cord blood of uncomplicated and post-kidney-transplant pregnancies and analyzed for their functional abilities with proliferation, cell migration, centrosome orientation and angiogenesis assays. Further, ECFCs from uncomplicated pregnancies were exposed to either umbilical cord serum from uncomplicated or post-kidney-transplant pregnancies.

**Results:**

Post-kidney-transplant ECFCs showed significantly less proliferation, less migration and less angiogenesis compared to control ECFCs. The presence of post-kidney-transplant umbilical cord serum led to similar functional aberrations of ECFCs from uncomplicated pregnancies.

**Conclusions:**

These pilot data demonstrate differences in ECFCs’ biological characteristics in offspring of women after kidney transplantation. Further studies are needed to monitor offspring’s long-term cardiovascular development and to assess possible causal relationships with immunosuppressants, uremia and maternal cardiovascular alterations.

**Impact:**

- Pregnancy after kidney transplantation has become more common in the past years but is associated with higher complications for mother and offspring.
- Little is known of the impact of maternal kidney transplantation and the mandatory immunosuppressive therapy on offspring vascular development.
- In this study we are the first to address and detect an impairment of endothelial progenitor cell function in offspring of kidney-transplanted mothers.
- Serum from post-transplant pregnancies also causes negative effects on ECFCs’ function.
- Clinical studies should focus on long-term monitoring of offspring’s cardiovascular health.

## Introduction

Since the first pregnancy resulted in a live birth following a kidney transplantation with consecutive immunosuppression in 1967, the number of female transplant recipients of child-bearing age has steadily increased and the issue of post-transplantation pregnancies has become considerably more important^1^. Due to modern immunosuppressive drugs, the risk of rejection has decreased and the fertility of women often restores after kidney transplantation^2^. Nevertheless, the management of these pregnancies is challenging and associated with higher rates of maternal and perinatal complications^3^.

Life-long immunosuppression is necessary to avoid graft rejection but might present a potential hazard for the offspring during pregnancy and afterwards. Most immunosuppressive drugs reach the fetal circulation by crossing the placental barrier^4^.

It is well-known that the intrauterine milieu and complications during pregnancy co-determine offspring’s health and cardiovascular risk. While exposure to adverse conditions, e.g. preeclampsia and diabetes, is associated with cardiovascular impairment in children in later life, data on offspring exposed to immunosuppressants *in utero* are limited and the impact of maternal transplantation and immunosuppressive therapy during pregnancy on offspring’s cardiovascular health has not been studied yet^5–14^.

Endothelial progenitor cells (EPCs) are impaired in several cardiovascular diseases^15–17^ and are considered as one of the strongest biomarkers to evaluate endothelial dysfunction and cardiovascular risk^18–21^. Endothelial colony-forming cells (ECFCs), a highly proliferative subgroup of EPCs, play an important role in angiogenesis and vascular repair and contribute to endothelial integrity^22,23^.

At the time of birth, cord blood-derived ECFCs are easily accessible and functional impairment has been reported in pregnancy complications which are associated with long-term cardiovascular impairment of the offspring^24–26^. In this study, we therefore tested whether fetal ECFCs are affected in pregnancies following maternal kidney transplantation.

## Materials and methods

### Patients

The study was approved by the Institutional Review Board of Hannover Medical School (approval no. 1443–2012 and 504–2009). Written informed consent was obtained from each participant.

Umbilical cord blood was collected from 12 uncomplicated and 6 post-kidney-transplant pregnancies for ECFC isolation, serum extraction or both. Uncomplicated pregnancies were defined as normotensive and without proteinuria, preexisting diabetes, hypertensive, vascular or renal disease, smoking or the use of illicit drugs ending with the delivery of a healthy baby. For the experiments, patients were matched by maternal age, BMI and gestational age at delivery.

### ECFC isolation, culture and characterization

Umbilical cord blood was collected into sterile EDTA-tubes immediately after delivery. Serum was extracted from separate serum tubes and stored at −80 °C until use for further experiments. ECFCs were isolated as previously described^25,27,28^ and cultured in endothelial growth medium 2 (EGM-2) consisting of endothelial basal medium (EBM-2; Lonza, Basel, Switzerland) supplemented with supplier provided supplements, 10% fetal bovine serum (FBS; Harvard Bioscience, Holliston, MA) and 1% penicillin/streptomycin (P/S; Bio&Sell, Feucht, Nürnberg, Germany) at 37 °C, 5% CO_2._ Day of appearance of ECFC colonies and total colony number were evaluated. ECFC colonies were noted as circumscribed cell monolayers with cobblestone-like morphology. Well-defined colonies were expanded and characterized by flow cytometry with typical phenotype markers (CD31 + , CD45-, and CD133-) by using appropriate antibodies (CD31, 130-117-390, BD Biosciences, San Jose, CA; CD45, 555483, BD Biosciences; and CD133, 130-090-826, Miltenyi Biotec, Bergisch Gladbach, Germany) and corresponding isotype controls (BD Biosciences, Miltenyi Biotec). ECFCs were used for experiments in cell culture passages 4–6.

### Cell impedance assay

For continuous monitoring of live cell proliferation, morphology and viability we used the xCelligence system (Roche, Basel, Switzerland), an impedance-based real-time analysis. The change in impedance is measured via the cell index, a dimensionless parameter reflecting cell adhesion, migration and proliferation and was calculated with the xCelligence Real-Time Cell Analyzer. The electrical impedance caused by adherent cells is converted into cell indices by the xCelligence software (v.1.2.1)^28^.

For ECFC comparison, 0.25 × 10^4^ cells from 4 control and 4 post-transplant ECFC lines were seeded in quadruplicates in EGM-2 with 10% FBS and 1% P/S onto a gold-coated E-Plate View 96-well plate (Roche) and then placed into the Real-Time Cell Analyzer SP station, positioned in a 37 °C incubator with 5% CO_2_ supply. Following adherence, the cell indices were aligned for all lines and then continuously monitored for 72 h.

To analyze serum effects, 0.25 × 10^4^ cells from 5 control ECFC lines were seeded in quadruplicates in EGM-2 with 2.5% FBS and 1% P/S and analyzed as described above. When a stable cell index was reached, 2.5% pooled control or pooled transplant serum were added to the cells. Cell impedance was recorded for 72 h.

### In vitro angiogenesis assay

To compare the capacity to form capillary tubule-like networks, 1.4 × 10^4^ cells/well from 4 control and 4 post-transplant ECFC lines were incubated in triplicates in 96-well plates pre-coated with 30 µL growth factor reduced Matrigel (BD Biosciences) for 14 h in EBM-2 with 5% FBS and 1% P/S^24^.

In separate experiments, 5 control ECFC lines were treated with either 5% control or 5% transplant serum in EBM-2 with 1% P/S. Phase contrast microscopic images were taken with a Leica DMI 6000 B microscope (Leica, Wetzlar, Germany). Total tube length and number of branch points in each visual field were calculated with ImageJ 1.50b (National Institutes of Health)^26^. Branch points were defined as nodes with connections to at least 3 tubes.

### Migration assay

To assess migration ability 5 × 10^4^ cells from 4 control and 4 post-transplant ECFC lines were seeded on gelatin-coated (Sigma-Aldrich, St. Louis, Missouri) wells of 6-well culture plates with EGM-2 containing 10% FBS and 1% P/S and grown to confluence. The cell monolayers were scratched with a sterile pipette tip to create a wound and washed with PBS. Afterwards, cells were cultured in fresh EBM-2 with 2.5% FBS and 1% P/S.

To analyze serum effects on ECFCs’ migration ability, 5 control ECFC lines were seeded, grown and scratched as described above. Then 5% control or transplant serum was added to the medium. Phase contrast microscopic images were immediately taken after scratching and then again after 18 h with a Leica DMI 6000 B microscope. Non-populated scratch areas were quantified by ImageJ 1.50b and subtracted to obtain the remigrated area.

### Centrosome orientation assay

In several cell types, there is a correlation between the position of the centrosome and the direction of cell movement: the centrosome is located behind the leading edge, suggesting that it serves as a control device for directional cell movement^29^. A change in the direction of cell movement precedes a re-orientation of the centrosome in the intended direction and is a sign of polarity of migrating cells^30^. During migration, the centrosome is positioned between the nucleus and the leading part, indicating the migrational status and direction^31^. To study differences in centrosome orientation, ECFCs were grown to confluence on coverslip glasses in 6-well culture plates and scratches were performed. After 1 h, cells were washed with PBS, fixed with 3% formaldehyde and 2% sucrose and permeabilized with 0.2% Triton X-100 (Sigma-Aldrich). Cells were incubated with an antibody against pericentrin (ab28144; Abcam, Cambridge, UK) in 2% normal goat serum (Thermo Fisher Scientific, Waltham, Massachusetts) and PBS for 2 h, washed 3x with PBS and incubated with Alexa Fluor anti-mouse IgG 546 (Thermo Fisher Scientific) for 2 h. Nuclear DNA was stained with 4′,6-diamidino-2-phenylindole (DAPI; Thermo Fisher Scientific), and coverslip glasses were mounted in mounting medium (ProLongGold; Thermo Fisher Scientific). Images were acquired randomly along the scratch with a Leica DMI 6000 B microscope (Leica).

### Statistical analysis

Normality distribution was tested by Shapiro-Wilk or D’Agostino normality test. Students paired *t* test was applied for serum associated data, students unpaired *t* test was used to analyze cell related data. Welch’s correction was administered in case of unequal variances. Not normally distributed data were assessed by Wilcoxon matched-pairs signed rank test or Mann Whitney test, respectively. Experimental data of biological replicates are presented as mean and standard error in the text. The obtained individual measured values (n) from each experiment were analyzed with Prism 9 (GraphPad Software, La Jolla, CA). P-values at < 0.05 were considered statistically significant and indicated in the figures as follows: * *p* < 0.05, ** *p* < 0.01.

## Results

### Patient characteristics

The kidney transplantation related clinical data for the transplanted women recruited for the study are given in Supplemental Table 1. The average time that has elapsed since transplantation was 5.5 years ± 1.5 years. All transplanted women received immunosuppression with tacrolimus. The mean tacrolimus plasma concentration was 5.4 µg/l ± 0.4 µg/l. All concentrations were in line with the average concentrations reported by Hebert et al.^32^

The comparison of pregnancy associated clinical and demographic data for women who provided umbilical cord blood are given in Table 1. Maternal age, gravidity, parity, maternal pre-pregnancy BMI, birth weight, birth weight percentile, delivery mode and sex of the baby were not statistically different between the control and the transplant group. The transplanted women had higher blood pressures at delivery (systolic: 144.7 ± 9.6 mmHg; diastolic: 87.3 ± 5.1 mmHg) compared to the control group (systolic: 112.9 ± 2.4 mmHg, *p* < 0.001; diastolic: 68.0 ± 1.6 mmHg, *p* = 0.004), although none had developed preeclampsia. The mean maternal serum creatinine concentration of the transplant group (Tx, 124 ± 11 µmol/l) was significantly higher than the control group (Con, 53 ± 3 µmol/l, *p* = 0.001) whereas the mean GFR was significantly lower (Tx 55 ± 9 ml/min/1,73 m^2^ vs. Con 123 ± 3 ml/min/1,73 m^2^, *p* < 0.001), respectively.

**Table 1:** Demographic and clinical characteristics of the study population who provided cord blood.

	Control group (*n* = 12)	Transplant group (*n* = 6)	*p* value
**Maternal characteristics**			
Maternal age (years)	29.50 ± 1.28	29.00 ± 1.98	0.83
Ethnicity			0.34
European	9 (75)	3 (50)	
Asian	3 (25)	3 (50)	
Primigravida	4 (30)	4 (67)	0.32
Primipara	8 (67)	6 (100)	0.25
Maternal pre-pregnancy BMI (kg/m^2^)	24.12 ± 1.25	27.22 ± 1.65	0.16
Gestational weight gain	15.36 ± 2.10	14.57 ± 4.31	0.85
Gestational SBP, pre-delivery (mmHg)	112.9 ± 2.4	144.7 ± 9.6	<0.001
Gestational DBP, pre-delivery (mmHg)	68.0 ± 1.6	87.3 ± 5.1	0.004
Gestational age at delivery (weeks)	37.49 ± 0.89	34.74 ± 1.79	0.18
Caesarean delivery	9 (75)	5 (83)	>0.99
Conception mode spontaneous	12 (100)	5 (83)	0.33
Gestational diabetes mellitus	0	0	NA
PIH or preeclampsia	0	0	NA
Tocolysis	2 (17)	1 (17)	>0.99
ASA prophylaxis	0 (0)	3 (50)	0.03
Creatinine (µmol/l)	53 ± 3	124 ± 11	0.001
GFR (ml/min/1,73 m^2^)	123 ± 3	55 ± 9	<0.001
**Neonatal characteristics**			
Birth weight (g)	3055 ± 228	2534 ± 418	0.25
Birth weight percentile	44.75 ± 7.34	52.00 ± 9.06	0.56
Sex of the baby male	8 (67)	4 (67)	>0.99
Preterm birth < 37 weeks	3 (25)	3 (50)	0.34
RDS prophylaxis	3 (25)	2 (33)	>0.99

Data are presented as mean ± standard error or number (% of total); *ASA* acetylsalicylic acid, *BMI* body mass index, *SBP* systolic blood pressure, *DBP* diastolic blood pressure, *GFR* glomerular filtration rate, *NA* not applicable, *PIH* pregnancy induced hypertension, *RDS* respiratory distress syndrome.

### Lower cell index of fetal ECFCs under transplant conditions

The time to the appearance of the first ECFC colony (Tx 8.5 ± 0.9 days vs. Con 9.2 ± 2.4 days, *n* = 4–5, *p* = 0.81) and the total number of colonies formed (Tx 9.0 ± 3.2 colonies vs. Con 13.4 ± 5.4 colonies, *n* = 4–5, *p* = 0.53) did not differ between the control and the transplant group.

**Fig. 1:**
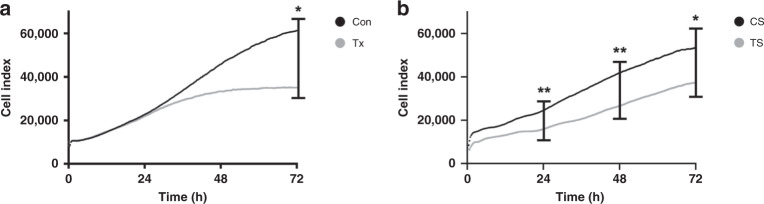
Lower cell index of fetal ECFCs under transplant conditions. **a** Overlay of growth curves of ECFCs from healthy or post-transplant pregnancies. The cell index of transplant ECFCs is significantly lower after 72 h. **b** Overlay of growth curves of ECFCs treated with control or transplant serum. The presence of transplant serum leads to a significantly lower cell index after 24, 48 and 72 h. *n* = 4–5; Con control, Tx transplant, CS control serum, TS transplant serum. * *p* < 0.05, ** *p* < 0.01.

The cell index of offspring from women with a kidney transplant, determined by real-time cell analysis, kept up with ECFCs derived from control umbilical cord blood in the first 24 h (Tx 22,149 ± 2,596 vs. Con 23,196 ± 1216, *n* = 4, *p* = 0.73), slowed down after 48 h (Tx 33,610 ± 8,250 vs. Con 46,468 ± 2,118, *n* = 4, *p* = 0.18) and showed a significantly lower increase after 72 h (Tx 35,240 ± 10,006 vs. 61,979 ± 4,164, *n* = 4, *p* = 0.049), Fig. 1a.

In the presence of transplant serum (TS) the cell index was markedly lower when compared to the incubation with serum from healthy controls (CS) (TS 16,209 ± 1,592 vs. CS 24,681 ± 1,871 after 24 h, *p* = 0.005; TS 27,470 ± 4,239 vs. CS 43,005 ± 5,986 after 48 h, *p* = 0.008; TS 38,962 ± 7,623 vs. CS 55,689 ± 10,260 after 72 h, *p* = 0.04, *n* = 5), Fig. 1b.

### Reduced tube formation ability of fetal ECFCs after maternal transplantation and after incubation with transplant cord serum

**Fig. 2:**
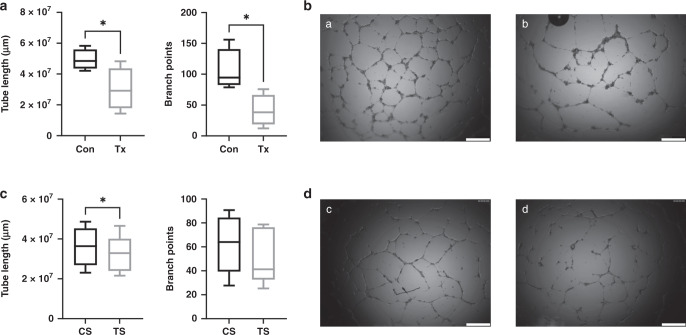
Reduced tube formation ability of fetal ECFCs after maternal transplantation and after incubation with transplant cord serum. **a** ECFCs of post-transplant pregnancies show less tube formation ability. **b** Representative images of control (a) and transplant (b) ECFCs. **c** Incubation with transplant serum impairs ECFCs’ angiogenesis. **d** Representative images of ECFCs treated with control (c) or transplant serum (d). Images were obtained after 14 h. Scale bar 500 µm. Box plots represent median, 25th and 75th percentile, whiskers the minimum and the maximum. *n* = 4–5. Con control, Tx transplant, CS control serum, TS transplant serum. * *p* < 0.05.

An in vitro angiogenesis assay was performed to reflect ECFCs’ ability to form de novo vessels in vivo. ECFCs derived from umbilical cord blood from transplant patients showed significantly lower tube lengths and lower number of branch points than ECFCs derived from umbilical cord blood from control patients (tube lengths: Tx 3.02 × 10^7^ µm ± 0.70 × 10^7^ µm vs. Con 4.93 × 10^7^ µm ± 0.34 × 10^7^ µm, *n* = 4, *p* = 0.048; branch points: Tx 41 ± 13 vs. Con 106 ± 17, *n* = 4, *p* = 0.02), Fig. 2a, b.

ECFCs’ ability to form capillary-like structures in Matrigel was significantly impaired in presence of umbilical cord serum from transplant patients in comparison to umbilical cord serum from healthy controls (TS 3.22 × 10^7^ µm ± 0.42 × 10^7^ µm vs. CS 3.62 × 10^7^ µm ± 0.44 × 10^7^ µm, *n* = 5, *p* = 0.047). The difference in the number of branch points did not reach significance (Tx: 52 ± 10 vs. Con 62 ± 11, *n* = 5, *p* = 0.30), Fig. 2c, d.

### Impaired migration of fetal ECFCs after maternal transplantation and after incubation with transplant cord serum

**Fig. 3:**
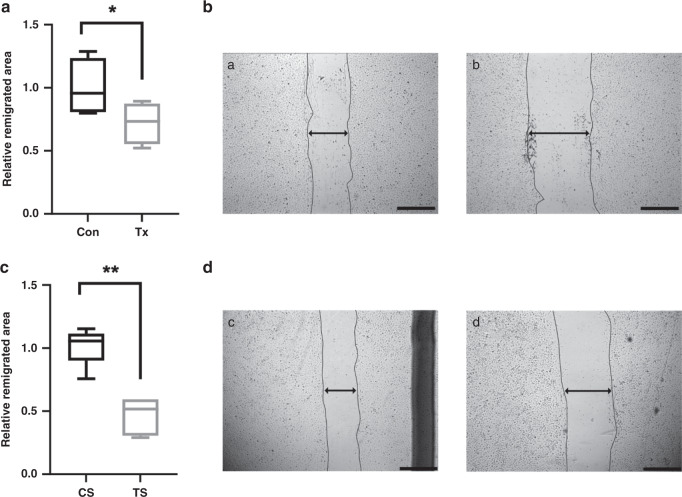
Impaired migration of fetal ECFCs after maternal transplantation and after incubation with transplant cord serum. **a** ECFCs from post-transplant pregnancies are less capable to migrate. **b** Representative images of control (a) and transplant (b) ECFCs after 18 h of migration. **c** Incubation with transplant serum impairs ECFC migration. **d** Representative images of ECFCs treated with control (c) or transplant serum (d). Scale bar 1000 µm. Box plots represent median, 25th and 75th percentile, whiskers the minimum and the maximum. Cell-free area after 18 h was subtracted from cell-free area at start to calculate remigrated area. Mean of control group was set to 1. *n* = 4–5. Con control, Tx transplant, CS control serum, TS transplant serum. * *p* < 0.05, ** *p* < 0.01.

We addressed the migration capacity of transplant patients’ offspring’s ECFCs in a scratch wound healing assay. Transplant ECFCs showed significantly less wound closure after 18 h than control ECFCs (relative remigrated area: Tx 0.72 ± 0.08 vs. Con 1.00 ± 0.12, *n* = 4, *p* = 0.045), Fig. 3a, b.

In presence of transplant serum, ECFCs remigrated about half as much of the area as when incubated with control serum (relative remigrated area: TS 0.46 ± 0.07 vs. CS 1.00 ± 0.07, *n* = 5, *p* = 0.001), Fig. 3c, d.

### Delayed pro-migratory positioning of fetal ECFCs from kidney transplanted women

**Fig. 4:**
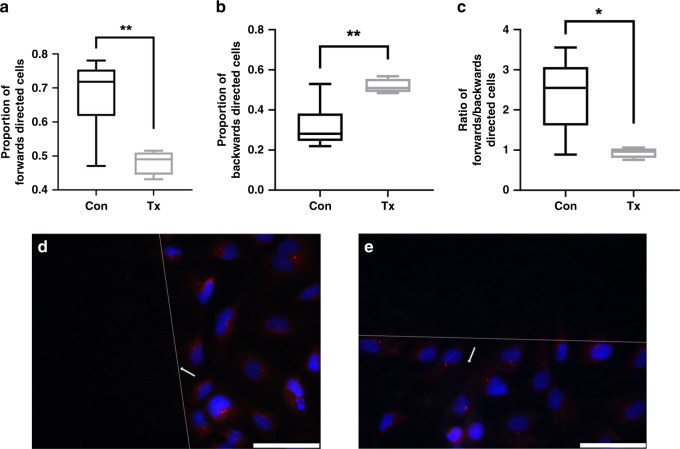
Delayed pro-migratory positioning of fetal ECFCs from kidney transplanted women. Centrosome localization is indicated by pericentrin staining (red) in immunofluorescence 1 h after the scratch performance. Nuclei were counterstained with DAPI (blue). Transplant ECFCs show less forwards (**a**) and more backwards (**b**) orientation than control ECFCs. Ratio of forwards and backwards orientation is calculated in **c**. Representative images of control and transplant ECFCs are shown in **d** and **e**. White lines indicate scratch borders. White arrows show the migration direction of the cells: forwards (towards the scratch) and backwards (away from the scratch). At least 37 cells were counted per line. Scale bar 50 µm. Box plots represent median, 25th and 75th percentile, whiskers the minimum and the maximum. *n* = 4–7, Con control, Tx transplant. * *p* < 0.05, ** *p* < 0.01.

Pericentrin staining was applied to gain insight in centrosome orientation. In accordance to the findings in the scratch wound healing assay, the proportion of ECFCs in direction towards the wound as well as the ratio of forwards and backwards directed cells were significantly lower in the transplant group compared to the control group (forwards: Tx 0.48 ± 0.02, *n* = 4 vs. Con 0.68 ± 0.04, *n* = 7, *p* = 0.006; backwards: Tx 0.52 ± 0.02, *n* = 4, vs. Con 0.32 ± 0.04, *n* = 7, *p* = 0.006; ratio: Tx 0.94 ± 0.06, *n* = 4, vs. Con 2.38 ± 0.34, *n* = 7, *p* = 0.01), Fig. 4.

## Discussion

In this study, we report a significant impairment of main biological characteristics of fetal ECFCs from mothers with a kidney transplant. A similar effect was observed when ECFCs derived from healthy pregnancies were exposed to umbilical cord serum from pregnancies after maternal kidney transplantation. To our knowledge, we are the first to address endothelial progenitor cells in transplant patients’ offspring as surrogate marker for vascular health.

The foundation for cardiovascular diseases in later life can already be laid during pregnancy^7,9^. Therefore, it is pivotal to identify risk factors for cardiovascular health as early as possible, when classical risk factors are not yet visible. This might pave the way for early intervention and primary prevention. Pregnancies after kidney transplantation carry a higher risk for the development of pregnancy complications, e.g. pregnancy-induced hypertension, preeclampsia, IUGR, and preterm birth^33^—well-known risk factors for future cardiovascular impairment of mothers and offspring^12,34–36^.

Fetal EPCs, which are considered to be involved in vascular homeostasis and repair^37^, appear to be an adequate model in this context to study vascular health. Our findings are in line with previous studies which describe an impairment of offspring ECFC number and function in gestational diseases or in the newborn period which are associated with later cardiovascular impairment of the progeny. In infants with bronchopulmonary dysplasia, a lung disease associated with prematurity, decreased numbers of ECFCs were reported^38^. In preeclampsia, a hypertensive disorder of pregnancy, the number of ECFC colonies was lower compared to controls^39^ and cells showed reduced proliferation, migrated less^40^ and formed fewer tubules^24^ which corresponds to our findings in transplant ECFCs. Recently, another study drew the link between ECFCs, neonatal complications and future cardiovascular diseases. It was demonstrated that in former preterms elevated systolic blood pressure significantly correlated with alterations in ECFC proliferation and tube formation^41^. These findings support our assumption that ECFCs are a suitable marker for vascular impairment.

We additionally demonstrated a negative effect on ECFCs derived from healthy pregnancies when incubated with cord serum of transplant pregnancies compared to cord serum of healthy controls. It seems possible that this observation may be the consequence of a substance circulating in the materno-placental-fetal system. The underlying disease that led to the transplantation is often associated with numerous cardiovascular risk factors and end organ damage, which only partially regress after the transplantation. The post-kidney-transplant cohort in our study still showed significantly higher concentrations of creatinine as an example for circulating urinary substances. In this context, EPCs have shown to be reduced in numbers, function and differentiation in chronic kidney disease patients as well as in uremia^42,43^. Another potential cause for our observations is the mandatory use of immunosuppressive agents, e.g. tacrolimus. Adverse effects include hypertension, hypercholesterinemia and hyperglycemia with the corresponding effects on the vascular system, leading to endothelial dysfunction^44^. Rabbits exposed to calcineurin inhibitors *in utero* were reported to be asymptomatic at birth, but presented hypertension, proteinuria and chronic kidney disease in adulthood implying possible long-term effects of intrauterine exposure to calcineurin inhibitors^14,45^. Regarding pregnancies in humans, it has been reported that, while trying to maintain target whole blood concentrations, dosage titration leads to a considerable increase of unbound tacrolimus concentrations, which is suggested to have important clinical implications^46^. Tacrolimus can accumulate in placenta as well as in ex vivo perfused placental tissue what could be associated with cytotoxic effects on placental level^47^. Unfortunately, the amount of serum obtained from umbilical cord was insufficient to record the concentration of tacrolimus or other metabolites in the newborns included in our study. However, we recently demonstrated calcineurin inhibitor induced functional impairment of ECFCs already in therapeutic concentrations^28^. These findings support our hypothesis of a possible contribution of immunosuppressive medication to reduced ECFC function in offspring of transplant patients. Apart from circulating substances themselves, there might also be a role for exosomes derived from endothelial or circulating cells. It has been shown that conditioned media or exosomes derived from ECFCs of patients with a known cardiovascular disease led to dysregulation of migration and impairment of tube formation of healthy ECFCs. It was stated that this effect is possibly mediated via the introduction of RNAs including miRNAs^48^.

In general, the pathogenesis of fetal complications is difficult to assess as there are many interacting factors such as the intrauterine exposure to immunosuppressive agents, higher incidence of preterm birth as well as the concomitant maternal pathologies like high blood pressure that can influence the fetal outcome^14,24,49^. Although not significant, mean gestational age was shorter in the transplant recipients in our study. This could have influenced the study results, but the literature is not clear on this. Baker et al. reported that preterm cord blood grew more ECFC colonies due to a higher proliferative capacity than term blood did and ECFCs had a similar angiogenic capacity^50^. This is in line with higher numbers of ECFC colonies in the study by Munoz-Hernandes et al.^39^ but in contrast to the findings by Ligi et al. who describe similar ECFC colony numbers but impaired function of preterm ECFCs^51^. It is worth noting that three out of six women in the transplant group have taken low-dose acetylsalicylic acid (ASA), which in pregnancy is used in the prevention of preeclampsia. In this context Hu et al. found a favorably impact on EPC migratory function and on the prevention of senescence using low-dose ASA^52^. At high-dose ASA they and Chen et al. observed an impairment of EPC function^52,53^. Considering the positive effect of low-dose ASA in clinical but also in in vitro studies on vascular and endothelial health one would expect this to be reflected in ECFC characteristics of the transplant cohort. However, the effects of the aberrant milieu in transplant recipients seem to mask the favorable impact of ASA in our study. Altogether, the history of kidney transplantation and the complications mentioned cannot be discussed independently of one another and should be understood as invitation for interdisciplinary thinking.

Although the number of pregnancies after kidney transplantation has increased, the single-center experience still remains quite low, leading to a small sample size in our pilot study. As in the latter we already detected considerable effects, we wish to share our results to encourage other researchers to contribute to the further elucidation of underlying mechanisms. Confirmatory studies including more participants are needed to corroborate our results and to better reflect the kidney transplant patients’ variety. Also, higher sample sizes would allow to adjust the results for the impact of potential confounders, e.g. gestational age at delivery. The transplant population of our study displayed heterogenous characteristics. For the six women included there were five different causes that led to the former kidney failure. Further, the time between transplantation and pregnancy differed from 2 to 10 years. The validity of our results is correspondingly limited. Another limitation of our study is reflected in the short assay time. Our in vitro analyses covered a period from 14 to 72 h, so no conclusions can be drawn about long-term effects.

So far, there are very few long-term follow-up studies targeting children of transplanted mothers. Most of the available information is limited essentially to classical parameters such as height, weight and head circumference monitoring which fortunately were unremarkable in the majority of children^14,54,55^. Nevertheless, as systemic alterations after intrauterine calcineurin inhibitor exposure were detected only in adult rodents^45^, Boulay et al. consequently concluded that the lack of symptoms in children might not be predictive of the absence of long-term effects^14^. Therefore, and considering the results of our study, further efforts are clearly needed to get a broader picture of possible consequences of maternal transplantation on their offspring’s cardiovascular health. Continuous multidisciplinary long-term follow-up studies should be implemented to open up the possibility for early interventions when facing cardiovascular risk.

## Supplementary information

Supplemental Table S1

## Data Availability

The datasets generated during and/or analyzed during the current study are available from the corresponding author on reasonable request.

## Category of study

Clinical study combined with basic science.
