# Supplementary material for: Fetal endothelial colony-forming cell impairment after maternal kidney transplantation
Source: Pediatr Res. 2022 Jun 22;93(4):810–7. doi: 10.1038/s41390-022-02165-x (PMC10033415; doi:10.1038/s41390-022-02165-x)
Supplement: Supplementary file 1 — Supplemental Table S1 [file 41390_2022_2165_MOESM1_ESM.pdf]

Supplemental Table S1. Clinical information of transplanted participants.

Patient	Underlying disease	Time since transplantation (years)	Therapeutic drug level (µg/l)*	Creatinine (µmol/l)*	GFR (ml/min/1,73 m2)*
1	Reflux nephropathy, interstitial nephritis	2	6.3	118	53
2	Glomerulonephritis	10	3.6	127	49
3	Glomerulonephritis	5	6.6	153	38
4	Nephronophthisis	10	5.4	136	47
5	Type I diabetes mellitus	2	5.6	135	45
6	unknown	4	5.0	74	100

* measured on the day of admission for delivery, GFR; glomerular filtration rate
